# Mitochondrial DNA mutations in head and neck cancer are infrequent and lack prognostic utility

**DOI:** 10.1038/bjc.2011.96

**Published:** 2011-03-22

**Authors:** C Challen, H Brown, C Cai, G Betts, I Paterson, P Sloan, C West, M Birch-Machin, M Robinson

**Affiliations:** 1 Centre for Oral Health Research, School of Dental Sciences, Newcastle University, Framlington Place, Newcastle-upon-Tyne NE2 4BW, UK; 2 Institute of Cellular Medicine, Newcastle University, Newcastle-upon-Tyne NE1 7RU, UK; 3 School of Cancer and Enabling Sciences, University of Manchester, Christie Hospital NHS Trust, Manchester M20 4BX, UK; 4 School of Life Sciences, University of Westminster, London W1B 2UW, UK

**Keywords:** head and neck squamous cell carcinoma, mitochondrial DNA, mutations, hypoxia

## Abstract

**Background:**

Mitochondrial DNA (mtDNA) mutations occur in head and neck squamous cell carcinoma (HNSCC) and are most frequently detected in the displacement-loop (D-loop) region. The D-loop is considered to be important because it controls mitochondrial gene expression and mtDNA replication. There is currently no evidence that mtDNA mutations can be used as prognostic or predictive biomarkers in HNSCC.

**Methods:**

We used denaturing high performance liquid chromatography to screen the entire mitochondrial genome of six oral squamous cell carcinoma-derived cell lines and then focused on detecting D-loop abnormalities in 34 HNSCC tissue samples.

**Results:**

Mitochondrial DNA mutations are not ubiquitous in HNSCC because only half of the cell lines had detectable mtDNA abnormalities following screening of the entire mitochondrial genome and only 18% (6 of 34) of tissue samples had D-loop mutations. There was no correlation between D-loop mutations and determinates of clinical outcome; specifically, tumour stage and the expression of hypoxia-inducible genes included in a highly prognostic hypoxia metagene.

**Conclusions:**

Taken together, these data suggest that mtDNA D-loop mutations are stochastic events that may not significantly influence the biology of HNSCC and supports the hypothesis that mtDNA mutations in cancer represent bystander genotoxic damage as a consequence of tumour development and progression.

Head and neck squamous cell carcinoma (HNSCC) is a major world health problem; estimates indicate that there are over 400 000 new cases diagnosed each year (Cancer Research UK, 2010). Typically, most patients present with advanced disease which has a poor prognosis and more than a third of the patients die within 5 years of diagnosis (Cancer Research UK, 2010). The search for prognostic and predictive biomarkers is a key strategy that could be used to improve the management of patients with HNSCC.

Somatic mutations in mitochondrial DNA (mtDNA) have been increasingly observed in human cancers and have been proposed as important oncological biomarkers (Jakupciak *et al*, 2005; Chatterjee *et al*, 2006). Mitochondrial DNA mutations have been detected in HNSCCs and their premalignant counterparts (Ha *et al*, 2002; Chatterjee *et al*, 2006; Mithani *et al*, 2007). Mutations occur throughout the mitochondrial genome, but are most frequently detected in the displacement-loop (D-loop) region; around a third of HNSCC samples harbour D-loop mutations (range 2–67% Table 1; Fliss *et al*, 2000; Sanchez-Cespedes *et al*, 2001; Tan *et al*, 2003; Lièvre *et al*, 2006; Pai *et al*, 2006; Prior *et al*, 2006; Zhou *et al*, 2006, 2007; Dasgupta *et al*, 2010). The D-loop region is considered to be important because it is the major control site for mtDNA expression and it is also involved in mtDNA replication (Taanman, 1999).

The biological significance of mtDNA mutations in cancer remains unclear (Chatterjee *et al*, 2006). Nevertheless, evidence is emerging that mtDNA damage has direct effects on the malignant phenotype and that these effects are mediated by reactive oxygen species (ROS; Petros *et al*, 2005; Ishikawa *et al*, 2008). Interestingly, mitochondrial ROS contributes to stabilisation of HIF-1α protein in hypoxia, leading to the proposal that ROS act as ‘oxygen sensors’ to trigger the hypoxia response (Klimova and Chandel, 2008). Recently, Sun *et al* (2009) showed expression of mutant mitochondrial NADH dehydrogenase 2 (ND2) in a HNSCC-derived cell line increased ROS production, which resulted in HIF-1α stabilisation and a shift to aerobic glycolysis. Furthermore, persistent increased levels of ROS, in the context of mutated mtDNA, may represent one of the mechanisms that drive genomic instability. Reactive oxygen species are DNA damaging agents that could perpetuate mtDNA damage and also cause genotoxic damage to the nucleosome (Lee and Wei, 2009). The link between mtDNA abnormalities, increased ROS and hypoxia may be particularly significant in HNSCC because of the association between hypoxia and an adverse prognosis in the disease (Nordsmark *et al*, 2005; Winter *et al*, 2007; Buffa *et al*, 2010).

In the present study, we screened the entire mitochondrial genome of six oral squamous cell carcinoma-derived cell lines for mtDNA deletions and mutations using denaturing high performance liquid chromatography (DHPLC) and then focused on identifying D-loop abnormalities in a set of HNSCC tissue samples. For the first time, we examined the relationship between mtDNA mutations and the expression of hypoxia-inducible genes in HNSCC tissue samples.

## Materials and methods

### Cell lines

Six human oral squamous cell carcinoma-derived cell lines were used (Table 2). Four of the cell lines have been described previously (Prime *et al*, 1990). The two new cell lines (H764A and H764B) were a kind gift of Professor S Prime (University of Bristol). HaCaT cells (Boukamp *et al*, 1990) and primary cultures of normal oral keratinocytes grown from explanted biopsy material were used as controls for high-throughput screening of cell line mtDNA. Cell line matched normal DNA was used to precisely characterise ‘screen-detected’ mtDNA abnormalities. Genomic DNA extracted from contiguous fibroblast cultures was available for four of the cell lines (H314, H357, H400, and H413; Drugan *et al*, 1997) and DNA extracted from formalin-fixed paraffin-embedded tissue (striated muscle) was used for H764A and H764B.

### Tissue samples

The tissue samples (tumour and normal mucosa) were derived from patients with previously untreated primary HNSCC. The research was given a favourable opinion from the Newcastle and North Tyneside 2 Research Ethics Committee (Reference 09/H0907/58). The clinical features of the samples are summarised in Table 3. The tissues were harvested at the time of surgical resection and were immediately placed in RNAlater (Applied Biosystems, Warrington, UK) before storage in liquid nitrogen.

### Preparation of DNA

Genomic DNA was extracted from cell pellets using a standard laboratory kit (QIAGEN Ltd, Crawley, UK). DNA was extracted from homogenised tissue samples using Tri Reagent (Sigma-Aldrich, Gillingham, UK) and cleaned using a standard laboratory kit (QIAGEN). Extracted DNA samples were diluted to 200 ng *μ*l^−1^ for use in downstream analysis.

### Long template PCR

Genomic DNA from the cell lines were screened for large-scale mtDNA deletions using the Expand Long Template PCR System as previously described (Roche Diagnostics Ltd, Burgess Hill, UK; Ray *et al*, 2000). DNA from a patient with mitochondrial myopathy, characterised by a 4976-bp mtDNA deletion, was used as a positive control (kind gift of Professor R Taylor, Newcastle University).

### Mitoscreen assay and DHPLC analysis

Genomic DNA samples were screened for small deletions, insertions, and point mutations using the Mitoscreen Assay System (Transgenomic Ltd, Glasgow, UK). The system comprises 19 overlapping primer sets that amplify the entire mitochondrial genome. Reactions were carried out according to the manufacturer's standard protocol. Briefly, PCRs were performed in 20–40 *μ*l reactions containing 100 ng of genomic DNA, 0.15 *μ*M of each primer and 2.5 units of Optimase polymerase (Transgenomic Ltd). In all, 15 of the 19 primer sets generated large amplicons that required digestion with restriction enzymes before heteroduplex formation. Heteroduplexed DNA was analysed by DHPLC on a Transgenomic Wave DNA Fragment Analysis System 3500 using Navigator software (Transgenomic Ltd).

### Automated sequencing

Denaturing high performance liquid chromatography fractions harbouring putative mtDNA abnormalities were eluted and dehydrated using a DNA concentrator (Gyrovap, Howe and Co., London, UK). DNA was resuspended in 5 *μ*l of water and reamplified using appropriate primer sets. The PCR products were resolved on a 2% low melting point agarose gel (NuSieve 3.1 Agarose, Lonza Rockland Inc., Rockland, ME, USA) containing ethidium bromide. The DNA band representing the amplicon was excised from the gel and the DNA was extracted using the QIAquick Gel Extraction Kit (QIAGEN). The purified DNA was eluted in 30 *μ*l of Tris/EDTA buffer. The sequencing reaction was carried out using the GenomeLab Dye Terminator Cycle Sequencing Kit (Beckman Coulter UK Ltd, High Wycombe, UK) according to the manufacturer's protocol. The products were run on a Beckman CEQ Genetic Analysis System 8000 (Beckman Coulter). Sequences were visualised using MacVector 7.0 software (Oxford Molecular Ltd, Oxford, UK) and compared with the mtDNA revised Cambridge reference sequence (MITOMAP, 2010).

### Hypoxia-inducible gene expression

Tissue samples were prepared for quantitative real-time PCR (qRT–PCR) as described previously (Winter *et al*, 2007). Quantitative real-time PCR for CA9, PGAM1, SLC2A1, and VEGFA was performed using Taqman assays (Applied Biosystems) on a low-density array format with normalisation to appropriate endogenous reference genes. Cycle threshold (Ct) values were converted to a measure of gene expression relative to the endogenous reference gene expression using the 2^−*Δ*Ct^ formula before statistical analysis as previously described (Livak and Schmittgen, 2001).

## Results

### Screening of cell lines for mtDNA abnormalities

None of the cell lines contained any large-scale mtDNA deletions (data not shown). Using DNA extracted from normal oral keratinocytes and HaCaT cells as a comparator, DHPLC profiles from the cell line DNA had a number of abnormalities. The abnormalities included broader wave profiles, peak shifts, and a change in the number of fragments in those samples subject to restriction enzyme digestion. Whole mitochondrial genome screening of the six cell lines generated 114 amplicons representing around 100 kb of DNA. A total of 17 mtDNA abnormalities were detected using this method and abnormalities were identified in all of the cell lines.

### Characterisation of cell line mtDNA abnormalities by sequence analysis

Sequencing of ‘screen-detected’ mtDNA abnormalities in the cell lines alongside matched normal DNA demonstrated that around half of the abnormalities (8 of 17) were common to both samples and represented mtDNA polymorphisms. H357, H400, and H413 had polymorphic mtDNA, but no somatic mtDNA mutations. By contrast, H314 had a complex mtDNA genotype with nine homoplasmic point mutations located in two regions of the mitochondrial genome, namely cytochrome oxidase I and the D-loop (Table 4). There were three base pair changes in the cytochrome oxidase I gene of H314 that were not present in the matched fibroblast DNA. The base changes were all synonymous and there was no predicted change in the amino-acid sequence of the codon. Six point mutations were detected in the D-loop; the matched fibroblasts followed the revised Cambridge reference sequence (MITOMAP, 2010). The base pair changes were all located in hypervariable segment 1 (HVS1) of the D-loop.

**Figure 1:**
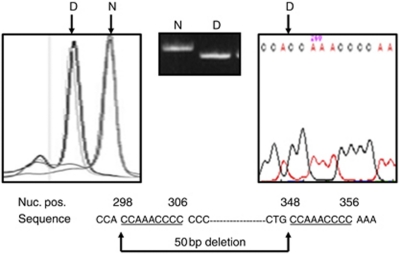
H764A and H764B cell lines harboured a homoplasmic 50 bp deletion in the D-loop region. The deletion (D) was identified by DHPLC, DNA electrophoresis, and DNA sequencing (from left to right, respectively; N=matched normal DNA sample). The deletion was located between nucleotide positions (nuc. pos.) 298/306 and 348/356; the breakpoints were flanked by a 9-bp repeat sequence 5′-CCAAACCCC-3′.

H764A and H764B, cell lines derived from the same patient representing a primary oral squamous cell carcinoma and a corresponding lymph node metastasis, respectively, had two sequence abnormalities not detected in the matched normal DNA. The first was a homoplasmic 50 bp deletion in the D-loop between nucleotide positions 298/306 and 348/356; the breakpoints were flanked by a 9-bp repeat sequence 5′-CCAAACCCC-3′ (Figure 1). The deletion was located in a non-coding, control region of the D-loop covering the mtTF1-binding site, replication primer, and conserved sequence blocks 2 and 3. An identical 50 bp deletion has previously been reported in four gastric cancers and a hepatocellular carcinoma (Burgart *et al*, 1995; Lee *et al*, 2004). The second sequence abnormality was a homoplasmic point mutation in the D-loop; G-A at nucleotide 16 129 in HVS1.

### Screening tissue samples for mtDNA abnormalities in the D-loop region

**Figure 2:**
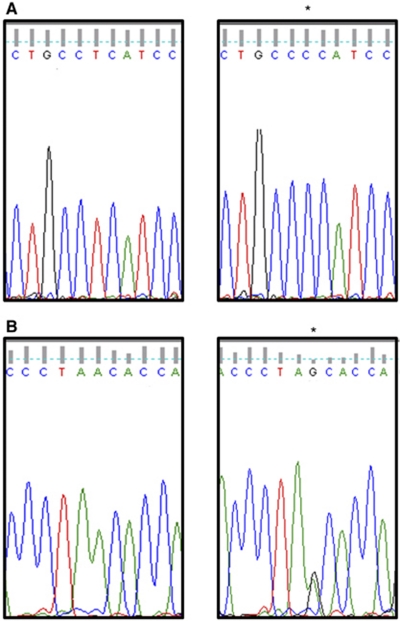
D-loop mutations in head and neck squamous cell carcinoma tissue samples. (**A**) Homoplasmic mutation at 146 T-C (^*^) in sample 87. Sequence shown from nucleotide position 141–151 for normal sample (left panel) and tumour sample (right panel). (**B**) Heteroplasmic mutation at 374 A-G (^*^) in sample 47. Sequence shown from nucleotide position 369–379 for normal sample (left panel) and tumour sample (right panel). In the tumour, there are two peaks at 374; there is a mixture of A and G nucleotides.

Twenty-two abnormalities were detected following screening of 102 amplicons in 34 HNSCC with matched normal mucosal samples. Sequencing of the variant amplicons demonstrated that 15 of the samples contained known sequence variants (polymorphisms) that were identical in the normal and tumour DNA (data not shown). Eight somatic mutations were detected in seven samples; sample 85 contained two mutations (Table 5). The majority (6 of 8) of the mutations were located in the D-loop; two were located in 12S rRNA gene. Eighteen percent (6 of 34) of the samples harboured a D-loop mutation and there was a mixture of homoplasmic and heteroplasmic mutations (Table 5; Figure 2). One of the D-loop mutations (146 T-C) has previously been reported in ovarian and prostate cancer (Figure 2A; MITOMAP, 2010).

### Relationship of D-loop mutations to clinical parameters and hypoxia-inducible genes

**Figure 3:**
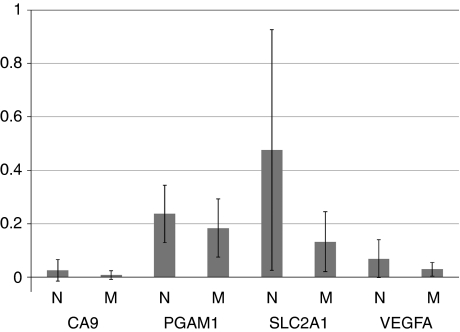
The mean expression of four hypoxia-inducible genes using qRT–PCR for samples with normal (N) and mutated (M) D-loop sequences.

The samples with D-loop mutations did not have any distinct clinico-pathological characteristics; there was no clear association with patient age, gender, tumour location, grade, and stage. Analysis of overall survival showed no difference for patients with D-loop mutations compared with those patients with normal D-loop sequences. We investigated the relationship between mtDNA D-loop mutations and the expression of four hypoxia-inducible genes included in a highly prognostic hypoxia metagene (CA9, PGAM1, SLC2A1, and VEGFA; Winter *et al*, 2007; Buffa *et al*, 2010). For each of the four genes, the samples with D-loop mutations (*n*=6) had lower mean gene expression levels when compared with those samples that had normal D-loop sequences (*n*=23), however, there was no statistically significant difference in hypoxia gene expression between the two groups (CA9 0.03 *vs* 0.01, *P*=0.30; PGAM1 0.24 *vs* 0.18, *P*=0.29; SLC2A1 0.48 *vs* 0.13, *P*=0.08; VEGFA 0.07 *vs* 0.03, *P*=0.19; parametric *t*-test, SPSS Statistics 17.0, SPSS Inc., Chicago, IL, USA; Figure 3).

## Discussion

In the current study, we screened oral squamous cell carcinoma-derived cell lines and HNSCC tissue samples for mtDNA mutations. Large-scale deletions of the mitochondrial genome have been reported in oral SCC (Tan *et al*, 2003; Shieh *et al*, 2004), however, our study and others (Zhou *et al*, 2007) have not been able to detect such abnormalities and therefore the significance of large-scale mtDNA deletions in HNSCC is unclear. We used DHPLC to screen samples for small-scale deletions, insertions, and point mutations. The majority of studies investigating mtDNA abnormalities in HNSCC have focused on small regions of the mitochondrial genome using PCR and sequencing (Fliss *et al*, 2000; Sanchez-Cespedes *et al*, 2001; Lièvre *et al*, 2006; Pai *et al*, 2006; Prior *et al*, 2006). Sequencing alone lacks sensitivity, because the technique only reliably identifies mutant species when they represent at least 25% of the amplicons in the sequencing reaction. By contrast, DHPLC is highly sensitive and detects mutations at very low thresholds; <5% (Birket and Birch-Machin, 2007). Denaturing high performance liquid chromatography also benefits from high specificity; identical DHPLC profiles between matched tumour and normal DNA effectively excludes mutation (Van den Bosch *et al*, 2000). The combination of DHPLC analysis and sequencing of enriched amplicon fractions is likely to have optimised mutation detection in our study.

We found that mtDNA mutations are not ubiquitous in HNSCC because half of the cell lines had no detectable mtDNA abnormalities following screening of the entire mitochondrial genome and only a small proportion (18%) of the HNSCC tissue samples had D-loop mutations. Pooled data from nine studies that looked for D-loop mutations in over 400 HNSCCs indicate that around a third of HNSCCs harbour D-loop mutations; however, there is wide variation between different studies (range 2–67% Table 1). The latter may reflect differences in the methods of mutation detection or the intrinsic genetic heterogeneity of HNSCCs. Significantly, our data are comparable with the largest series examined to date (*n*=109; Lièvre *et al*, 2006), which detected D-loop mutations in only 21% of the samples.

The D-loop is considered to be particularly important because it controls mitochondrial gene expression and it is involved in mtDNA replication (Taanman, 1999). More recently, there is evidence that the D-loop has a functional role in the formation of mitochondrial nucleoids and the organisation of mtDNA during segregation and replication (He *et al*, 2007; Holt *et al*, 2007). Of the mutations detected in the current study, the majority were T-C, G-A base transitions, which are associated with oxidative damage (Fliss *et al*, 2000; Zhou *et al*, 2007) and raise the possibility that ROS have a pivotal role in perpetuating mtDNA damage. While, elevated levels of ROS are known to stabilise HIF-1α and trigger the hypoxia response (Klimova and Chandel, 2008), we were unable to demonstrate a correlation between mtDNA mutations and hypoxia-inducible gene expression.

The 50-bp deletion identified in the D-loop of both H764A and its corresponding lymph node metastasis, H764B, has previously been reported in gastric adenocarcinomas and hepatocellular carcinomas (Burgart *et al*, 1995; Lee *et al*, 2004). Furthermore, this small-scale deletion was also present in the metastases of two of the gastric cancers (Burgart *et al*, 1995). These findings are consistent with the concept of clonal expansion and conservation of mtDNA mutations during tumour progression. The functional significance of the deletion is unknown; however, one possible mechanism is that the deletion affects nucleoid formation, by interfering with ATAD3 binding, leading to altered mtDNA copy number (Holt *et al*, 2007). Increased mtDNA copy number has been described in HNSCCs (Kim *et al*, 2004) and is also associated with increased oxidative stress (Lee and Wei, 2009). These factors may account for the accumulation of the deletion to homoplasmic levels and its persistence during tumour progression.

There are a number of studies which indicate that D-loop mutations have prognostic significance. Studies in non-small cell lung cancer, colorectal cancer, and breast cancer showed D-loop mutations are associated with poor prognosis (Matsuyama *et al*, 2003; Lièvre *et al*, 2005; Tseng *et al*, 2006). The only study in HNSCC found no correlation between D-loop mutations and prognosis or response to chemotherapy in 109 patients (Lièvre *et al*, 2006). Similarly, in the present study, there was no obvious correlation between D-loop mutations and prognostic indicators, specifically, tumour stage and the expression of hypoxia-inducible genes included in a highly prognostic hypoxia metagene (Winter *et al*, 2007; Buffa *et al*, 2010). These data are consistent with other studies in a variety of other cancers (oesophagus, gastric, lung, and ovarian), showing no correlation between mtDNA abnormalities with clinico-pathological features, which limits their utility as prognostic biomarkers in these diseases (Lee and Wei, 2009).

In conclusion, our study indicates that mtDNA mutations are infrequent in HNSCC, but most commonly located in the D-loop region. Displacement-loop mutations are likely to be stochastic events (i.e., subject to the variation of chance) that may not significantly influence the biology of HNSCC. Our data support the hypothesis that mtDNA mutations in cancer represent bystander genotoxic damage as a consequence of tumour development and progression (Taylor and Turnbull, 2005).

## Figures and Tables

**Table 1:** Prevalence of D-loop mutations in head and neck squamous cell carcinoma

**Study**	**No. of patients**	**No. with D-loop mutations**	**Percentage %**
Fliss *et al* (2000)	13	3	23
Sanchez-Cespedes *et al* (2001)	51	19	37
Tan *et al* (2003)	18	12	67
Lièvre *et al* (2006)	109	23	21
Pai *et al* (2006)	67	41	61
Prior *et al* (2006)	30	16	53
Zhou *et al* (2006)	7	3	43
Zhou *et al* (2007)	83	24	29
Dasgupta *et al* (2010)	50	1	2
			
Total	428	142	33

**Table 2:** Derivation of the human oral squamous cell carcinoma-derived cell lines (Prime *et al*, 1990)

**Cell line**	**Age**	**Sex**	**Site**	**Grade**	**Stage**
H314	82	M	FOM	MD	II
H357	74	M	T	WD	I
H400	55	F	AP	MD	II
H413	53	F	BM	MD	II
H764A	70	M	FOM	MD	IV
H764B	70	M	LN	MD	IV

Abbreviations: AP=alveolar process; BM=buccal mucosa; FOM=floor of mouth; LN=lymph node; T=tongue; MD=moderately differentiated; WD=well-differentiated squamous cell carcinoma.

**Table 3:** The clinical features of squamous cell carcinoma tissue samples

**Sample no.**	**Age**	**Sex**	**Site**	**Grade**	**Stage**
34	73	M	Oral cavity	MD	IVA
35	65	M	Hypopharynx	MD	IVA
38	60	F	Oral cavity	MD	IVA
41	38	M	Larynx	MD	IVA
42	71	M	Hypopharynx	MD	IVA
43	58	M	Hypopharynx	Basaloid	IVA
44	68	F	Oral cavity	MD	II
45	68	M	Oral cavity	MD	II
46	44	M	Oropharynx	Basaloid	IVA
47	69	M	Oral cavity	PD	IVA
51	59	M	Oral cavity	MD	I
54	58	M	Larynx	PD	IVA
59	38	F	Oral cavity	MD	III
60	64	M	Oral cavity	MD	I
61	44	M	Oral cavity	MD	II
62	70	M	Larynx	NR	IVA
63	59	M	Oropharynx	Basaloid	IVA
64	54	M	Oropharynx	Basaloid	IVA
65	70	M	Larynx	MD	II
67	56	M	Oral cavity	MD	IVA
68	64	M	Larynx	MD	IVA
70	66	M	Oropharynx	Basaloid	IVA
71	53	M	Oral cavity	MD	III
73	55	M	Hypopharynx	MD	III
75	76	F	Larynx	MD	IVA
76	43	M	Oral cavity	MD	II
78	53	M	Larynx	MD	IVA
80	63	F	Larynx	MD	IVA
82	63	M	Larynx	PD	IVA
83	56	M	Larynx	PD	IVA
85	57	F	Oral cavity	MD	IVA
87	54	M	Oral cavity	MD	II
88	61	F	Oral cavity	MD	IVA
89	69	M	Hypopharynx	MD	IVA

Abbreviations: MD=moderately differentiated; PD=poorly differentiated; NR=not recorded.

**Table 4:** Mitochondrial DNA mutations in H314 cells

**Mitochondrial gene/region**	**D-loop region**	**Nucleotide position**	**mtDNA rCRS**	**Control H314 fibroblasts**	**Tumour H314**	**Mutation status**	**Reported previously^a^**
COI	NA	6152	T	T	C	HM	No
COI	NA	6179	G	G	A	HM	No
COI	NA	6180	G	G	A	HM	No
D-loop	HVS1	16 064	T	T	C	HM	No
D-loop	HVS1	16 093	T	T	C	HM	No
D-loop	HVS1	16 094	T	T	C	HM	No
D-loop	HVS1	16 102	T	T	A	HM	No
D-loop	HVS1	16 124	T	T	G	HM	No
D-loop	HVS1	16 145	G	G	A	HM	No

Abbreviations: COI=cytochrome oxidase I (np 5904–7445); D-loop=displacement-loop (np 16024–576); HVS1=hypervariable segment 1; NA=not applicable; mtDNA rCRS=mitochondrial DNA revised Cambridge reference sequence (MITOMAP, 2010); HM=homoplasmic.

a Mutation reported on a publically available database (MITOMAP, 2010).

**Table 5:** Mitochondrial mutations detected in head and neck squamous cell carcinoma tissue samples

**Case**	**Mitochondrial gene/region**	**D-loop region**	**Nucleotide position**	**mtDNA rCRS**	**Control tissue**	**Tumour**	**Mutation status**	**Reported previously^a^**
43	D-loop	HVS2	153	A	A	G	HM	No
47	D-loop	H-strand origin	374	A	A	A/G	HT	No
64	12S rRNA	NA	796	G	G	A	HM	No
83	D-loop	HVS2	265	T	T	T/A	HT	No
85	12S rRNA	NA	779	T	T	T/G	HT	No
85	D-loop	HVS1	16 129	G	A	G/A	HT	No
87	D-loop	HVS2	146	T	T	C	HM	Ovarian carcinoma Prostate tumour
88	D-loop	HVS1	16 145	G	G	G/A	HT	No

Abbreviations: HVS1/2=hypervariable segment 1/2; NA=not applicable; mtDNA rCRS=mitochondrial DNA revised Cambridge reference sequence (MITOMAP, 2010); HM=homoplasmic; HT=heteroplasmic.

a Mutation reported on a publically available database (MITOMAP, 2010).
